# An Intelligent Pressurized Thigh Band for Muscular Assistance and Multi-Mode Activity Recognition

**DOI:** 10.3390/s26051502

**Published:** 2026-02-27

**Authors:** Wenda Wang, Wenbin Jiang, Yang Yu, Wei Dong, Hui Dong, Yongzhuo Gao, Dongmei Wu, Weiqi Lin

**Affiliations:** State Key Laboratory of Robotics and System, Harbin Institute of Technology, Harbin 150001, China; wangwenda@stu.hit.edu.cn (W.W.); 2022110256@stu.hit.edu.cn (W.J.); 24s008036@stu.hit.edu.cn (Y.Y.); dongwei@hit.edu.cn (W.D.); dongh@hit.edu.cn (H.D.); gaoyongzhuo@hit.edu.cn (Y.G.); wdm@hit.edu.cn (D.W.)

**Keywords:** soft robot, sEMG, motion recognition, wearable assistive device

## Abstract

This study aims to develop a “sensing-actuation integrated” intelligent pressurized thigh band to assist the quadriceps, indirectly alleviate knee joint load, and achieve high-precision recognition of movement modes. The system comprises a portable integrated controller and a textile-integrated flexible pneumatic actuator. Experiments were conducted to evaluate the effects of different air bladder pressure conditions on metabolic rate and muscle activity. Simultaneously, pneumatic data corresponding to six common activities were collected, and a lightweight deep learning model was developed to enable high-precision motion classification. Finally, the model was deployed to an embedded platform to demonstrate its application potential. Results indicate that appropriate air bladder pressure significantly reduces quadriceps muscle activation and average metabolic cost. Furthermore, the deep learning model achieved 99.17% accuracy in recognizing the six activities and was successfully deployed to the embedded platform. This study validates the effectiveness of the intelligent pressurized thigh band in improving locomotor performance under static pressures and demonstrates the potential of air bladder pressure variations as a proxy indicator for movement intent for future closed-loop control.

## 1. Introduction

Globally, the issue of insufficient physical activity among adults is becoming increasingly severe. According to the 2022 “Global status report on physical activity” by the World Health Organization (WHO), approximately one in four adults (1.4 billion) worldwide do not meet the recommended levels of physical activity, which significantly increases the risks of non-communicable diseases and musculoskeletal injuries [1]. Although global organizations are actively promoting physical activity, this increase in exercise has been accompanied by a rising incidence of sports-related injuries. Research indicates that knee injuries constitute a significant proportion of such cases, particularly in running and high-intensity training [2]. As a central weight-bearing joint, the knee endures loads several times body weight and is highly susceptible to injury due to improper form or overtraining [3]. Therefore, developing intelligent assistive technologies to protect the knee, enhance performance, and reduce injury risks has become a critical issue in sports science and rehabilitation engineering. Exoskeleton technology offers an effective technological approach [4]; thus, the development of lightweight and intelligent knee assistive technologies holds significant scientific value and market prospects.

**Figure 1:**
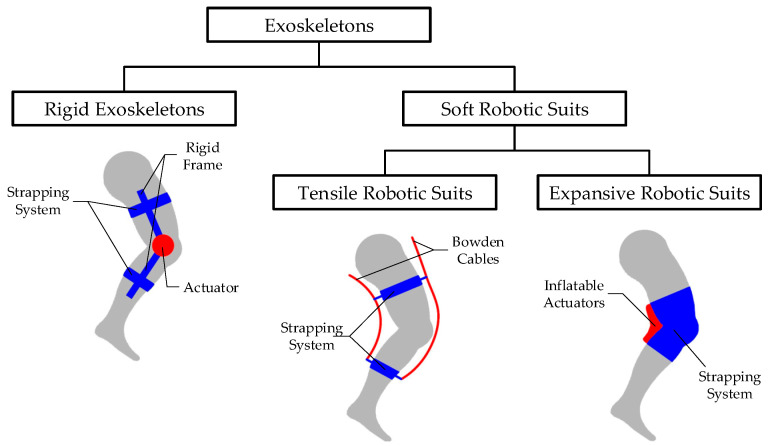
Schematic diagram of exoskeleton classification. In the figure, the red parts represent the drive units, the blue parts represent the other units, and the gray parts represent the human lower limb.

As shown in Figure 1, current knee assistive technologies primarily fall into two categories: exoskeletons and braces. Exoskeletons can be further divided into rigid exoskeletons and soft robotic suits. Rigid exoskeletons are typically designed to provide assistance using the simplest possible mechanical joints [5,6]. However, these fixed-center joints inevitably cause joint misalignment during movement [7]—that is, the inconsistency between the human and machine rotation centers. When joint misalignment occurs, the single mechanical rotation model of the knee joint can easily lead to joint dislocation [8]. Unlike rigid exoskeletons, soft exoskeletons, or soft robotic suits, usually lack rigid frame structures capable of bearing compressive loads [9]. Consequently, their inherent compliance allows them to conform to human anatomy, effectively eliminating the issue of joint misalignment. Based on the driving structure, soft robotic suits are typically classified into two types: tensile and expansive. Tensile Robotic Suits usually employ motors to pull Bowden cables, thereby driving anchor points strapped to the limbs to move the joints, as seen in studies [10,11]. While these soft components effectively address joint misalignment, they rely heavily on friction between the skin and the straps to transmit force; consequently, anchor point slippage can render assistance ineffective [12], overly tight binding can cause discomfort to the wearer [13], and may even lead to nerve and vascular damage [14]. The other category, Expansive Robotic Suits, such as those in [15,16], typically utilize pneumatic interference actuators (PIA) to generate torque by inflating air bladders to support human joints. However, due to their slow dynamics caused by inflation and deflation processes, such devices often cannot keep pace with the complete gait cycle. Furthermore, the forces transmitted by soft exoskeletons are characterized by uncertainty, which is detrimental to control [17]. Knee braces usually refer to prophylactic or functional knee braces. These braces have positive effects on knee stability [18] and related muscle control [19], and can reduce the risk of sports injuries [20]; however, they only provide limited support to the knee joint and do not fundamentally alleviate muscle fatigue. Knee stability relies heavily on the surrounding muscles, especially the quadriceps [21]. Therefore, directly assisting the muscles rather than the joint itself may be a more efficient and natural assistance strategy.

Furthermore, intelligent assistive devices require not only execution capabilities but also effective perception of the user. Compared to Inertial Measurement Units (IMUs), surface electromyography (sEMG) offers the advantage of detecting movement onset prior to physical motion [22]. However, its practical application is hindered by the need for meticulous skin preparation and high susceptibility to interference from sweat and motion artifacts. Some studies have attempted to use combinations of air bladders and pressure sensors to mimic certain sEMG functions. For instance, study [23] integrated air bladder pressure signals with IMUs, while study [24] utilized multiple pressure sensors for pattern recognition. However, these approaches often rely on multi-sensor fusion or complex configurations. Therefore, a simpler and more user-friendly sensing method is required to ensure practical applicability.

In summary, while rigid exoskeletons provide robust support, their inherent mechanical structures do not align with the complex kinematics of the human knee, often leading to joint misalignment. Conversely, soft exoskeletons address the issue of joint misalignment through compliant materials, yet they introduce challenges related to binding discomfort and control difficulties during dynamic movement. Traditional knee braces, on the other hand, offer only passive support and lack the capacity to actively alleviate muscle fatigue. Regarding intent perception, although sEMG is considered an ideal technology for its ability to capture movement intent in advance, its practical application is hindered by stringent skin preparation requirements and susceptibility to signal interference from sweat. Similarly, existing air bladder pressure sensor systems often rely on multi-sensor fusion or complex configurations, which increases system complexity and hinders practical application in dynamic scenarios.

To address these challenges, we propose a novel “sensing-actuation integrated” intelligent pressurized thigh band. Positioned at the distal thigh, this device delivers pneumatic assistance to the quadriceps, thereby indirectly alleviating the load on the knee joint. This approach mitigates common issues in joint-level exoskeletons, such as joint misalignment and anchor point slippage induced by reaction forces. Furthermore, the system is characterized by its light weight, low cost, and user-friendly nature, rendering it particularly suitable for use during dynamic exercise and daily workouts. We experimentally validated the device’s assistive capability via sEMG and metabolic power measurements; results demonstrated that appropriate air bladder pressure significantly reduced quadriceps muscle activation and average metabolic cost. Furthermore, we hypothesize that dynamic pressure variations inside the bandage during movement can serve as a novel, low-cost proxy for muscle contraction and movement intent. To validate this hypothesis, we collected pressure data across six common activities and developed a lightweight deep learning model, achieving high-precision classification of these locomotion modes. Finally, we deployed the model on an embedded platform (STM32), demonstrating its feasibility for practical deployment.

The innovations of this study are summarized as follows:

1. Integrated sensing-actuation design. The circumferential thigh air bladder serves a dual function as both a sensor and an actuator. This enables the simultaneous monitoring and assistance of multiple key muscle groups, offering a new paradigm for the design of intelligent assistive devices.
2. Multi-muscle group monitoring via a single sensor. Using a single air bladder pressure sensor, the system effectively captures contraction and expansion information from multiple key muscle groups. This approach simplifies the system architecture while reducing both cost and complexity.
3. Muscle-based assistance rather than joint-based assistance. By assisting the muscles directly, this approach avoids common issues associated with joint-level exoskeletons, such as joint misalignment and strap slippage. This not only improves user experience and safety but also better aligns with human biomechanics, resulting in a more natural assistance effect.

## 2. Materials and Methods

### 2.1. Hardware Design

**Figure 2:**
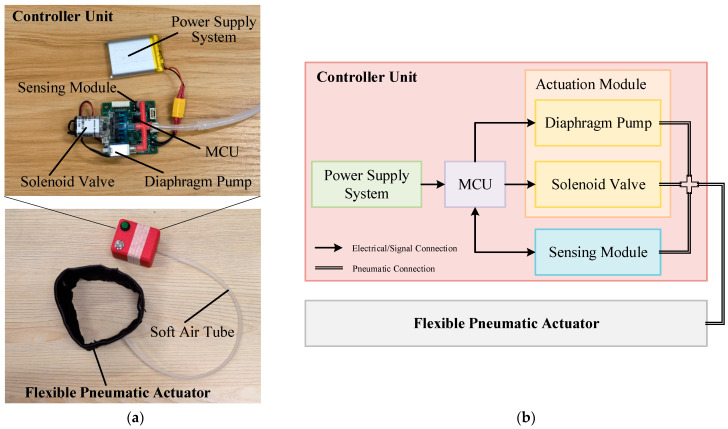
Hardware system schematic. (**a**) Photograph of the hardware system. (**b**) Schematic block diagram of the hardware system. In the figure, solid lines with arrows represent electrical or signal connections, while hollow lines represent pneumatic connections.

The hardware system is physically divided into two distinct yet interconnected modules: a portable integrated controller and a textile-integrated flexible pneumatic actuator. These two modules are connected via a soft air tube with an inner diameter of 3 mm for air transmission, as shown in Figure 2.

#### 2.1.1. Controller Unit

The controller unit serves as the control center and pneumatic source for the entire system. Its printed circuit board (PCB) is custom-designed and integrates the following key components:

At the core lies an STM32F407IGH6 microcontroller. This MCU was selected for its powerful ARM Cortex-M4 core, which features a maximum clock speed of 168 MHz and a built-in Hardware Floating Point Unit (FPU). This capability is crucial for the efficient execution of sensor data acquisition and processing algorithms, as well as for supporting the deployment of lightweight deep learning models on embedded devices.

Pressure monitoring is handled by a MEMS pressure sensor (MCP-H10, AID Information, Jining, China). Based on a piezoresistive sensing element, this sensor integrates internal signal amplification and multi-stage temperature compensation, capable of providing highly linear and stable output signals within the required pressure range.

The pneumatic system consists of a diaphragm pump (EDZP02, Kamoer, Shanghai, China) and a normally open two-way solenoid valve. The pump is characterized by low power consumption (1.5 W) and quiet operation (<55 dB), making it highly suitable for wearable application scenarios. The use of a normally open solenoid valve constitutes a reliable safety design; it ensures that the air bladder automatically deflates and depressurizes in the event of an unexpected power loss, thereby preventing user discomfort or potential issues.

The entire unit is powered by a 1000 mAh lithium-ion battery with a rated voltage of 4.2 V, supporting an estimated continuous operation of over 6 h.

#### 2.1.2. Flexible Pneumatic Actuator

This section consists of a customized air bladder and an integrated strap, with the core objective of achieving comfortable, safe, and efficient force transmission during human–robot interaction.

The force source of the actuator is a customized Thermoplastic Polyurethane (TPU) air bladder. TPU was selected for its excellent elasticity, wear resistance, and air tightness. The bladder is designed as a rectangular structure ($l_{1}$ is 30 mm, $l_{2}$ is 40 mm) and is formed into a loop by connecting the ends during application. This size and shape are intended to cover the primary regions of the entire thigh muscle group, thereby providing uniform assistive force while avoiding excessive compression on local blood vessels and nerves [14]. When fully inflated, the bladder can be approximated as a cylinder with a cross-sectional diameter (D) of 20 mm and a gas volume ($V_{b}$) of approximately 94,248 mm^3^.

(1) $$V_{b}=\frac{\pi D^{2}l_{1}}{4}$$

Such a muscle-bladder model was established in Ref. [24]. In their model, the muscle is assumed to be incompressible, connected to an elastic tendon with an elastic coefficient of k; each muscle is approximated as a cylinder with a uniform cross-sectional area and a constant volume; the total cross-sectional area of the air bladder and the corresponding muscle group remains constant, i.e.,

(2) $$A_{b}+A_{m}=\left(A_{b}+dA_{b}\right)+\left(A_{m}+dA_{m}\right)$$

where $A_{b}$ is the cross-sectional area of the air bladder and $A_{m}$ is the cross-sectional area of the corresponding muscle group, while $dA_{b}$ and $dA_{m}$ represent their respective variations. Based on this model, they calculated that

(3) $$f_{m}=\frac{kA_{b}x}{P_{b}A_{m}}dP_{b}$$

where $f_{m}$ is the muscle force transmitted through the tendon, x is the initial muscle length, $P_{b}$ is the initial air bladder pressure, and $dP_{b}$ is the variation in air bladder pressure. According to the model described above, k, $A_{b}$, x, $P_{b}$ and $A_{m}$ are all constants. Therefore, it can be derived that $f_{m}\propto dP_{b}$, indicating that variations in air bladder pressure can reflect muscle activity.

**Figure 3:**
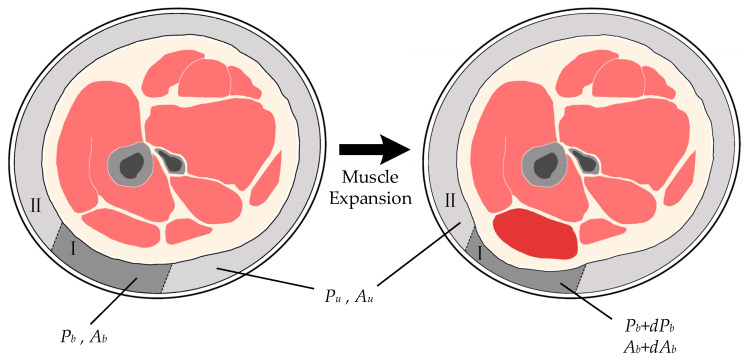
Improved muscle-bladder model established.

Considering that the air bladder covers multiple muscle groups, we incorporate the following assumptions into the muscle-bladder model established in Ref. [24], as shown in Figure 3. Let $P_{t}$ be the initial pressure of the entire air bladder. At any given instant, we assume that only one target muscle group undergoes contraction or expansion, while the state changes of the remaining muscle groups are negligible. Based on this assumption, the bladder system can be divided into two sub-regions at the moment of contraction: Region I corresponds to the contracting muscle group, with an initial cross-sectional area $A_{b}$ and initial pressure $P_{b}$, and variations $dA_{b}$ and $dP_{b}$, respectively; Region II corresponds to the remaining non-contracting muscle groups, with an initial cross-sectional area $A_{u}$ and initial pressure $P_{u}$.

Since these two sub-regions are interconnected through the internal space of the air bladder, pressure equalizes rapidly. Consequently, after a very short transient process, the pressure throughout the system tends to be uniform, reaching a new equilibrium state of $P_{t}+dP_{t}$ collectively. This process is assumed to be isothermal rather than adiabatic. This assumption is justified by the fact that the thin-walled bladder is in close contact with the human skin, which acts as a constant temperature heat source. The thermal equilibrium time is significantly shorter than the motion period, allowing the gas temperature to remain constant during the pressure variation. Consequently, this process follows the Ideal Gas Law

(4) PV=nRT

That is

(5) $$P_{b}A_{b}=\left(P_{b}+dP_{b}\right)\left(A_{b}+dA_{b}\right)$$

(6) $$P_{t}\left(A_{b}+A_{u}\right)=\left(P_{t}+dP_{t}\right)\left[\left(A_{b}+dA_{b}\right)+A_{u}\right]$$

From Equations (5) and (6), we obtain

(7) $$dA_{b}=-\frac{dP_{b}\left(A_{b}+dA_{b}\right)}{P_{b}}\approx -\frac{A_{b}}{P_{b}}dP_{b}$$

(8) $$dA_{b}=-\frac{dP_{t}\left[\left(A_{b}+dA_{b}\right)+A_{u}\right]}{P_{t}}\approx -\frac{A_{b}+A_{u}}{P_{t}}dP_{t}$$

Combining these two equations yields

(9) $$dP_{b}=\frac{P_{b}\left(A_{b}+A_{u}\right)}{P_{t}A_{b}}dP_{t}$$

Substituting Equation (9) into Equation (3) yields

(10) $$f_{m}=\frac{k\left(A_{b}+A_{u}\right)x}{P_{t}A_{m}}dP_{t}$$

According to the bladder-muscle model, k, $A_{b}+A_{u}$, x, $P_{t}$ and $A_{m}$ are all constants. Therefore, it can be derived that $f_{m}\propto dP_{t}$, indicating that variations in the overall bladder pressure can reflect muscle activity.

To comfortably secure the air bladder to the thigh, the TPU bladder is firmly encapsulated between two layers of highly elastic nylon fabric to form an integrated wearable unit. The strap design features adaptive fitting and anti-slip fixation. The inner and outer elastic fabric layers provide structural strength while ensuring a snug fit against the user’s leg circumference. Additionally, the inner surface of the strap is distributed with silicone anti-slip particles, which work synergistically with the inflated bladder to effectively prevent strap slippage during movement.

### 2.2. Experimental Design

**Figure 4:**
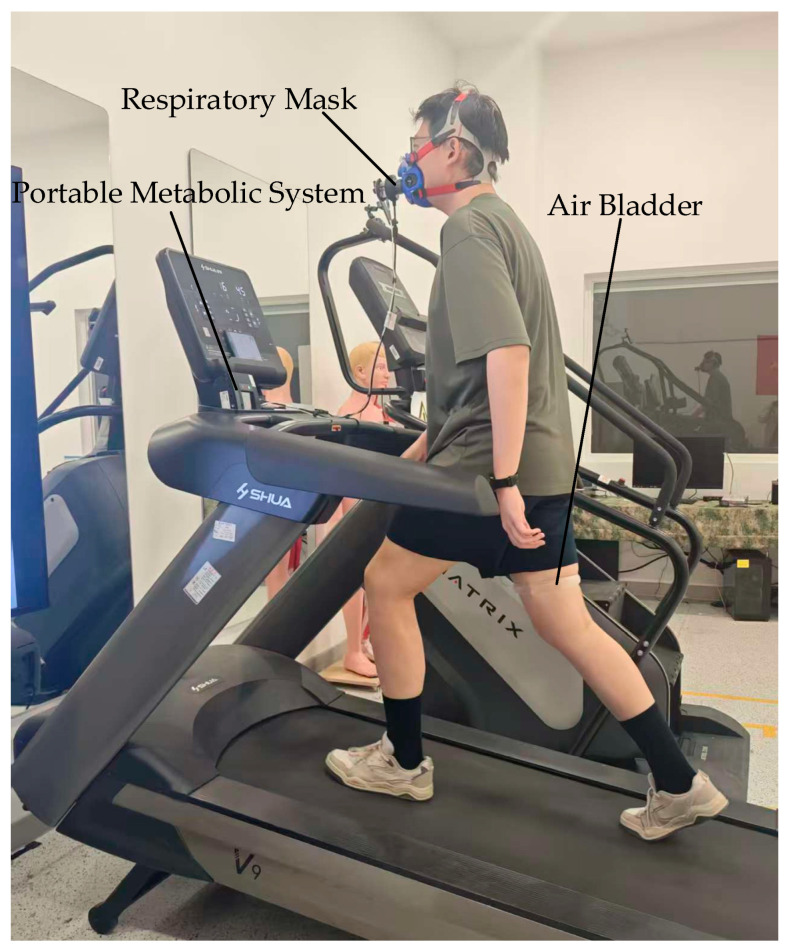
Figure of the experimental procedure. The scene shows a participant wearing the intelligent band on the right leg during the uphill experiment.

To verify the effectiveness of the intelligent pressurized thigh band, nine healthy male participants (age: 26.90 ± 2.41 years; height: 1.77 ± 0.04 m; weight: 85.78 ± 5.41 kg; mean ± standard deviation) were recruited for a series of experiments, as shown in Figure 4. The study protocol was reviewed and approved by the Clinical Trial Ethics Committee of Heilongjiang Provincial Hospital (protocol code 2022-153JY2), and all procedures were conducted in accordance with the Declaration of Helsinki. Prior to data collection, all participants received a detailed explanation of the study’s purpose and procedures and provided written informed consent. To ensure participants were familiarized with the device and to minimize learning effects, a familiarization session was conducted two days before the formal experiment. During this session, the working principle of the intelligent thigh band and controller parameters were introduced in detail, and participants were given ample time to adapt to wearing and operating the device.

The formal experiment employed a within-subjects design, in which each participant was required to complete six predefined motor tasks under three bladder pressure conditions (no bladder, 20 kPa, and 30 kPa). The motor tasks included static standing, walking (1.25 m/s), jogging (2 m/s), uphill walking (15% gradient, 1.25 m/s), stair ascent, and squatting. Specifically, the squatting task consisted of 3 sets of 10 repetitions, with a 5 min rest interval between sets, whereas each of the other tasks lasted for 5 min. To prevent interference from muscle fatigue, a 15 min rest interval was scheduled between the different motor tasks. To evaluate system performance while ensuring physiological safety, we selected two pressure levels: 20 kPa and 30 kPa. The determination of this range was based on both technical and physiological considerations. Technically, our preliminary tests indicated that pressures below 20 kPa resulted in poor contact stability and degraded signal quality; thus, 20 kPa was established as the lower threshold. Physiologically, we followed literature guidelines for non-invasive lower limb pressure [25] and pilot study feedback. Although 40 kPa was tested in the pilot study, it induced a noticeable sense of constriction in some participants. Consequently, 30 kPa was set as the upper limit to balance effective support with user comfort and safety [13].

Building upon the pressure safety limits, we also integrated redundant safety mechanisms into the hardware design to ensure user well-being during operation. Specifically, the pneumatic circuit utilizes normally-open valves, ensuring the airway remains open to the atmosphere in the event of a power failure, thus guaranteeing passive safety. Additionally, the connection between the air circuit and the bladder is designed for easy manual disconnection, allowing for rapid removal in emergencies. These design choices ensure that participants could halt the intervention instantly if any discomfort occurred.

To systematically evaluate the physiological effectiveness of the intelligent pressurized thigh band, comprehensive tests on cardiopulmonary function and muscle activity were conducted on the participants. Metabolic rate was measured using a portable metabolic system (K5, Cosmed, Rome, Italy), with the average oxygen uptake (VO2) and carbon dioxide production (VCO2) calculated over the final 2 min of each task. To obtain net metabolic cost, all data were normalized to the participants’ body weight and subtracted by their basal metabolic rate (BMR), which was determined from measurements taken during the static standing task. Simultaneously, wireless sEMG electrodes (PicoX, Cometa, Guangzhou, China) were used to collect electromyography signals from the rectus femoris (RF) to quantify muscle activity levels. The raw EMG signals underwent the following processing steps: first, maximal voluntary contraction (MVC) normalization was performed; next, a 20–500 Hz band-pass filter was applied to remove noise; this was followed by full-wave rectification; and finally, a 6 Hz low-pass filter was applied to obtain the linear envelope.

To establish the dataset for motion recognition, the air pressure data within the air bladder of the band were synchronously collected and stored at a constant sampling frequency during all physiological evaluation tasks. Since the mode and intensity of each motor task were strictly controlled, the acquired pressure data naturally corresponded to specific motion states. This high-quality, motion-labeled pressure data constituted the training dataset, which was used to train the deep learning model, enabling it to accurately identify and classify different motion states based on pressure fluctuation patterns.

It is important to note that the experimental design adopted in this study follows a modular validation paradigm. Specifically, the physiological effectiveness of pneumatic assistance and the accuracy of motion recognition are evaluated independently, rather than as a fully integrated closed-loop system. This strategy is designed to rigorously establish the reliability and feasibility of the two fundamental modules—perception and execution—prior to their integration. By validating the distinct benefits of pressure-based assistive effects and the robustness of the pressure-based recognition model, this work provides the essential theoretical basis and empirical evidence required to underpin a future adaptive, closed-loop control strategy.

### 2.3. Data Analysis Methods

All statistical analyses were performed using GraphPad Prism 10.4.1 software. Data are expressed as mean ± standard deviation. To evaluate the effect of bladder pressure configurations on physiological indicators, a one-way repeated measures ANOVA was employed. The factor for the ANOVA was the bladder pressure configuration (no bladder, 20 kPa, 30 kPa). When the ANOVA results were significant, Tukey’s post hoc test was performed for pairwise comparisons. The significance level for all tests was set at α = 0.05.

## 3. Results

### 3.1. Physiological Effectiveness Validation

The physiological analysis presented herein is grounded in data collected across six motion states. Static standing was incorporated solely as a baseline reference for normalizing metabolic costs. Additionally, the deep squatting task was omitted from the metabolic analysis due to its transient nature; the short duration of each set precluded the stabilization of respiratory signals. Consequently, while sEMG metrics encompass the five dynamic movement states, the metabolic results are confined to the four tasks that satisfied the criteria for steady-state data acquisition.

**Figure 5:**
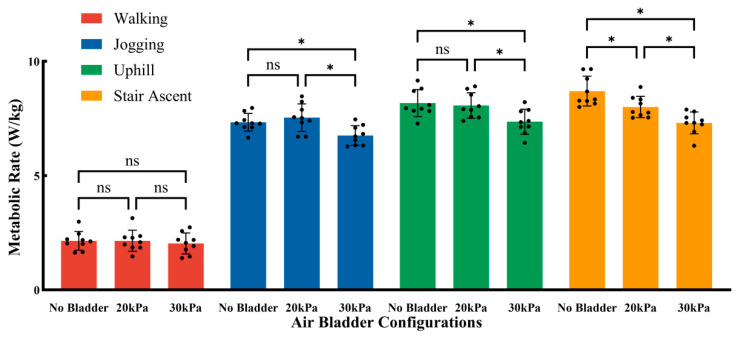
The effects of three air bladder pressure configurations on metabolic rate across different motion modes. The black dots indicate the individual raw data points. Error bar indicates standard deviation (SD). Asterisk (*) denotes a significant difference, ns indicates no significant difference.

Figure 5 illustrates the effects of three air bladder pressure configurations (no bladder, 20 kPa, and 30 kPa) on metabolic rate across different motion modes. Under the walking condition, there were no significant differences in metabolic rate among the different bladder pressure configurations; compared with the no bladder configuration, neither the 20 kPa nor the 30 kPa pressure resulted in significant changes in metabolic rate. Under the jogging condition, there was no significant difference in metabolic rate between the no bladder and 20 kPa configurations; however, compared with the no bladder configuration, the 30 kPa bladder pressure significantly reduced net metabolic rate (by 0.58 ± 0.23 W/kg, *p* = 0.048). Similarly, under the uphill walking condition, there was no significant difference in metabolic rate between the no bladder and 20 kPa configurations; compared with the no bladder configuration, the 30 kPa bladder pressure significantly reduced net metabolic rate (by 0.82 ± 0.27 W/kg, *p* = 0.001). Under the stair ascent condition, both the 20 kPa and 30 kPa configurations significantly reduced net metabolic rate (20 kPa: reduced by 0.69 ± 0.25 W/kg, *p* = 0.030; 30 kPa: reduced by 1.39 ± 0.25 W/kg, *p* < 0.001); furthermore, the effect of the 30 kPa configuration was significantly superior to that of the 20 kPa configuration (30 kPa vs. 20 kPa: reduced by 0.70 ± 0.25 W/kg, *p* = 0.029).

**Figure 6:**
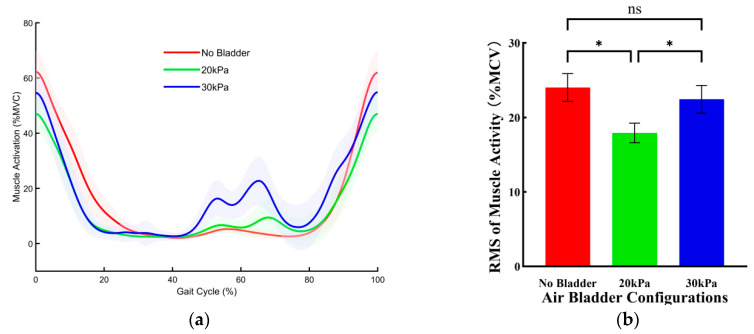
The effects of three bladder pressure configurations on the muscle activity of the RF during walking. (**a**) Changes in RF muscle activation over a single gait cycle across different air bladder configurations. Error bands indicate SD. (**b**) The RMS of RF muscle activation. Error bar indicates SD. Asterisk (*) denotes a significant difference, ns indicates no significant difference.

Figure 6 illustrates the effects of three bladder pressure configurations on the muscle activity of the rectus femoris (RF) during walking. Compared with the no bladder configuration, the 20 kPa bladder pressure configuration significantly reduced muscle activity, whereas the 30 kPa configuration had no significant effect on muscle activity. Compared with the no bladder configuration, the root mean square (RMS) of muscle activity under the 20 kPa configuration was reduced by 6.11 ± 0.80%MVC (*p* < 0.001).

**Figure 7:**
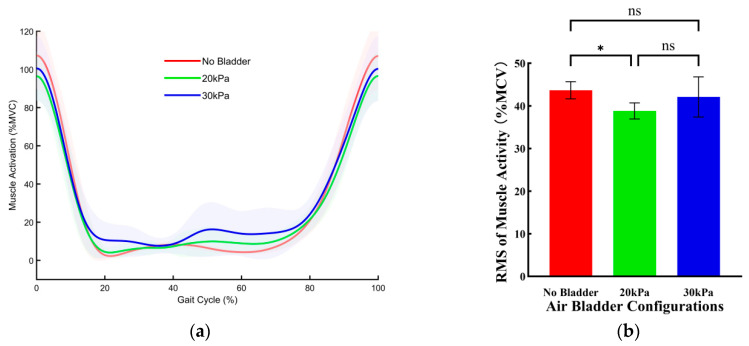
The effects of three bladder pressure configurations on the muscle activity of the RF during jogging. (**a**) Changes in RF muscle activation over a single gait cycle across different air bladder configurations. Error bands indicate SD. (**b**) The RMS of RF muscle activation. Error bar indicates SD. Asterisk (*) denotes a significant difference, ns indicates no significant difference.

Figure 7 illustrates the effects of three bladder pressure configurations on the muscle activity of the RF during jogging. Compared with the no bladder configuration, the 20 kPa bladder pressure configuration significantly reduced muscle activity, whereas the 30 kPa configuration had no significant effect on muscle activity. Specifically, compared with the no bladder configuration, the RMS of muscle activity under the 20 kPa configuration was reduced by 4.84 ± 1.50%MVC (*p* = 0.008).

**Figure 8:**
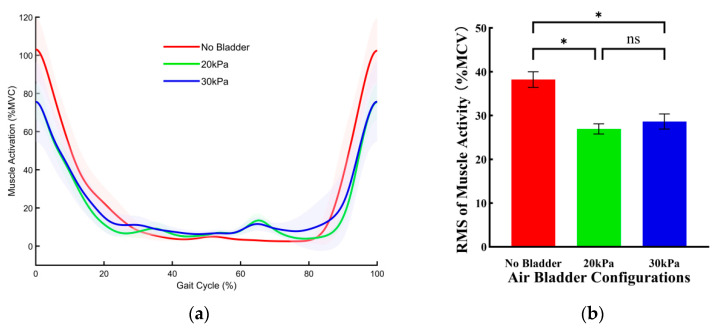
The effects of three bladder pressure configurations on the muscle activity of the RF during uphill walking. (**a**) Changes in RF muscle activation over a single gait cycle across different air bladder configurations. Error bands indicate SD. (**b**) The RMS of RF muscle activation. Error bar indicates SD. Asterisk (*) denotes a significant difference, ns indicates no significant difference.

Figure 8 illustrates the effects of three bladder pressure configurations on the muscle activity of the RF during uphill walking. Compared with the no bladder configuration, both the 20 kPa and 30 kPa configurations significantly reduced muscle activity. Specifically, compared with the no bladder configuration, the RMS of muscle activity was reduced by 11.27 ± 0.75% MVC (*p* < 0.001) under the 20 kPa configuration and by 9.59 ± 0.75%MVC (*p* < 0.001) under the 30 kPa configuration. There was no significant difference in effect between the 20 kPa and 30 kPa configurations.

**Figure 9:**
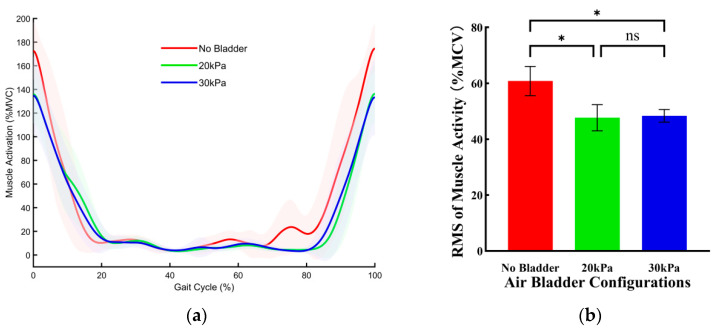
The effects of three bladder pressure configurations on the muscle activity of the RF during stair ascent. (**a**) Changes in RF muscle activation over a single gait cycle across different air bladder configurations. Error bands indicate SD. (**b**) The RMS of RF muscle activation. Error bar indicates SD. Asterisk (*) denotes a significant difference, ns indicates no significant difference.

Figure 9 illustrates the effects of three bladder pressure configurations on the muscle activity of the RF during stair ascent. Compared with the no bladder configuration, both the 20 kPa and 30 kPa configurations significantly reduced muscle activity. Specifically, compared with the no bladder configuration, the RMS of muscle activity was reduced by 13.15 ± 2.01%MVC (*p* < 0.001) under the 20 kPa configuration and by 12.46 ± 2.01%MVC (*p* < 0.001) under the 30 kPa configuration. There was no significant difference in effect between the 20 kPa and 30 kPa configurations.

**Figure 10:**
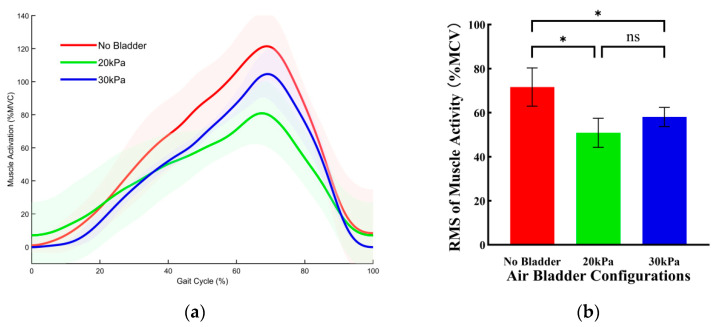
The effects of three bladder pressure configurations on the muscle activity of the RF during squatting. (**a**) Changes in RF muscle activation over a single gait cycle across different air bladder configurations. Error bands indicate SD. (**b**) The RMS of RF muscle activation. Error bar indicates SD. Asterisk (*) denotes a significant difference, ns indicates no significant difference.

Figure 10 illustrates the effects of three bladder pressure configurations on the muscle activity of the RF during squatting. Compared with the no bladder configuration, both the 20 kPa and 30 kPa configurations significantly reduced muscle activity. Specifically, compared with the no bladder configuration, the RMS of muscle activity was reduced by 20.76 ± 3.19%MVC (*p* < 0.001) under the 20 kPa configuration and by 13.56 ± 2.82%MVC (*p* < 0.001) under the 30 kPa configuration. There was no significant difference in effect between the 20 kPa and 30 kPa configurations.

### 3.2. Motion Recognition Performance

To achieve real-time, low-latency recognition of user motion intent, we designed and implemented a deep learning system fully deployed on a microcontroller. Taking a single pressure sensor as input, the system utilizes an STM32 microcontroller to complete the entire process of data acquisition, model inference, and control decision-making. This edge computing architecture eliminates the dependence on external processing units, ensuring system autonomy and rapid response capability [26], which serves as the core technical support for the smart strap to achieve precise assistance.

#### 3.2.1. Data Acquisition and Preprocessing

The training and testing data for this study were obtained through a dedicated data acquisition platform. The core of this platform is an STM32 microcontroller that samples analog-to-digital converter readings from the pressure sensor at a rate of 100 Hz (every 10 ms) and transmits the calculated pressure values to a personal computer via serial communication in real-time. Data collection was conducted during the physiological effectiveness verification experiments, covering six typical daily activities and motion modes: standing, walking, jogging, uphill walking, stair ascent, and squatting. After acquiring raw data for all motion modes, we applied a unified labeling and preprocessing pipeline.

To ensure data integrity, we first conducted a visual inspection of the raw pressure waveforms. We observed that for the squatting task, the pressure waveform during the rest period between squats closely resembled the waveform of standing. In fact, these segments correspond to the subject being in a standing position. To prevent this standing data from being mislabeled as part of the squatting activity, we implemented a preprocessing step. Specifically, we filtered out all pressure values below 20 kPa for the 20 kPa bladder configuration and below 30 kPa for the 30 kPa bladder configuration. This step effectively isolated the active squatting segments from the passive standing periods.

**Figure 11:**
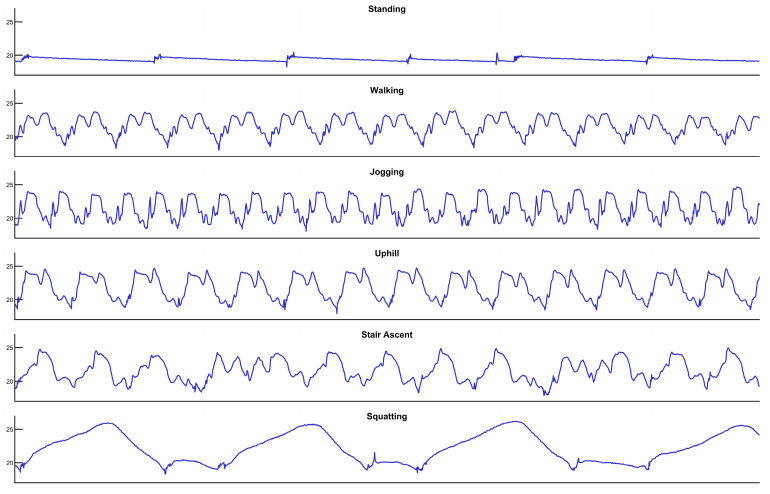
Characteristic pressure waveforms for six distinct motion states (standing, walking, jogging, uphill walking, stair ascent, and squatting) over a fixed duration. The waveform for standing is relatively stable, while dynamic movements (walking, jogging, uphill, stair ascent) exhibit distinct periodic oscillations. The squatting waveform shows a large, slow pressure surge, which is markedly different from the rapid fluctuations of other tasks. These visual differences confirm the intrinsic separability of the sensor data.

An example of the raw pressure data, illustrating the distinct patterns for each motion state, is presented in Figure 11. This figure shows 15 s of data from a single participant during the experiment with the 20 kPa bladder configuration.

#### 3.2.2. Dataset Partitioning

To ensure the unbiasedness and effectiveness of model training, we constructed a balanced dataset. By upsampling or downsampling data from various categories, we ensured that the sample sizes for the six motion categories (standing, walking, jogging, uphill walking, stair ascent, and squatting) were exactly equal. Ultimately, the entire dataset was partitioned into a training set and a testing set. The training set consisted of a total of 30,300 samples (5050 per class) for model parameter learning and optimization; the testing set consisted of a total of 1200 samples (200 per class) for the performance evaluation of the final model.

#### 3.2.3. Model Architecture

We designed a lightweight hybrid neural network with an architecture aimed at efficiently extracting spatiotemporal features from 500-millisecond pressure time series. The network structure specifically includes:

The input pressure data was first standardized using Z-score normalization. The dataset was then partitioned into training and testing sets with a ratio of 8:2, utilizing stratified sampling to maintain class distribution consistency. The network structure specifically includes:

- Feature Extraction Layer. A one-dimensional convolutional layer with 48 filters and a kernel size of 4, used to capture local pressure patterns, followed by a max pooling layer with a pool size of 2 to reduce dimensionality.
- Temporal Modeling Layer. Two LSTM layers (with 24 and 12 units, respectively) equipped with a Dropout rate of 0.3 to prevent overfitting, used to learn long-term dependencies in time series.
- Classification Layer. A fully connected layer with 6 nodes using the Softmax activation function, outputting probabilities corresponding to the six motion categories.

**Figure 12:**
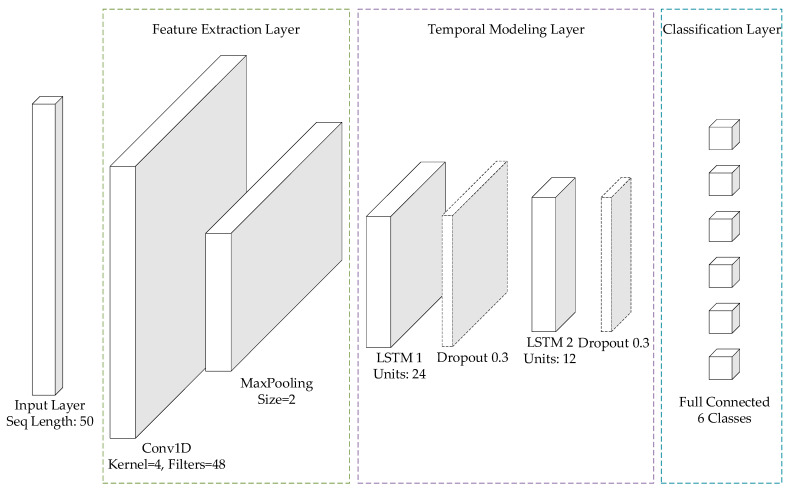
Schematic diagram of the CNN-LSTM hybrid neural network structure.

For the training process, the model was compiled using the Adam optimizer and trained for 300 epochs with a batch size of 128, minimizing the categorical cross-entropy loss function. The model has a total of 9104 parameters and a model file size of approximately 35.57 KB, fully meeting the requirements for embedded deployment. The schematic of the model is shown in Figure 12.

#### 3.2.4. Performance Evaluation

**Figure 13:**
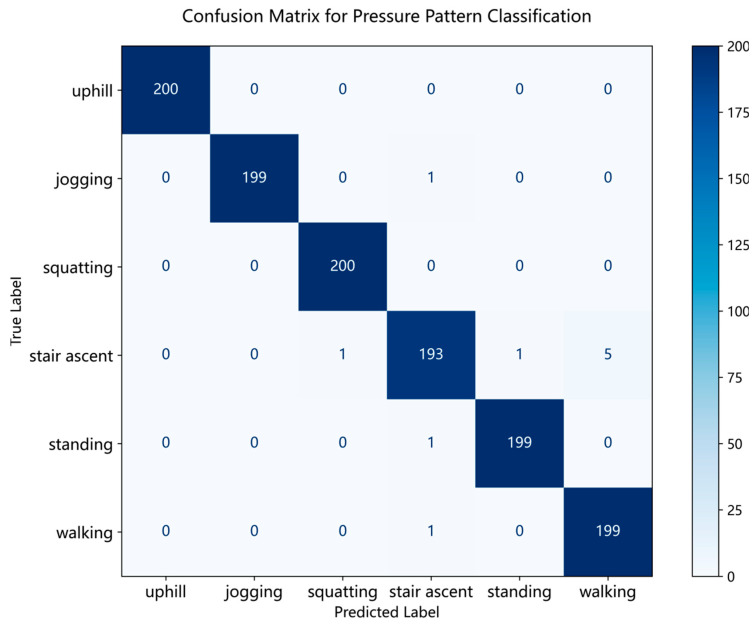
Confusion matrix of the proposed model on the test set.

The model demonstrated excellent classification performance on the independent testing set, achieving an overall accuracy of 99.17%, as shown in the confusion matrix in Figure 13. Furthermore, as shown in Table 1, the classification accuracy for jogging, uphill walking, squatting, and standing all exceeded 99%, and the recall rate for each category was above 96.5%. These results strongly demonstrate that our proposed lightweight model can achieve high-precision motion recognition while maintaining extremely low computational overhead.

### 3.3. Performance Evaluation of Embedded Model

Benefiting from its streamlined network architecture, the trained model can be directly deployed without the need for compression. We utilized the STM32Cube.AI development toolkit for code generation and performance analysis. This lightweight model exhibits extremely low resource occupancy on the target STM32F4 platform: the entire AI system (including model weights and runtime libraries) occupies only 57.7 KiB of Flash memory (total capacity 1024 KiB) and 9.9 KiB of runtime memory (total capacity 192 KiB). The model has a computational complexity of 216,564 MACC (Multiply-Accumulate Operations). Given the 168 MHz system clock, the theoretical inference time is approximately 3.87 ms. Practical deployment tests using the STM32Cube.AI profiler further confirmed a measured latency of approximately 4 ms. This minimal delay lays a solid hardware foundation for low-latency real-time motion recognition.

## 4. Discussion

The results of the physiological effectiveness verification experiments demonstrate that the intelligent pressurized thigh band can produce significant biomechanical benefits under specific bladder pressure settings. Table 2 details the assistive effects of the smart strap. In terms of metabolic rate, a bladder pressure of 30 kPa reduced the net metabolic rate during jogging, uphill walking, and stair ascent by 7.8%, 10.0%, and 16.0%, respectively, indicating a trend where the assistive effect becomes more pronounced as exercise intensity increases. In terms of muscle activity, the pressure of 20 kPa exhibited superior performance, reducing the RMS value of the RF by 11.1% to 29.5% during activities such as slow walking, jogging, uphill walking, stair ascent, and squatting. This significant assistive effect exceeded our initial expectations.

To investigate the underlying mechanism, we drew an analogy between the function of the smart strap and the physiological principles of compression garments. Existing studies indicate that applying appropriate pressure to muscles can restrict non-functional vibrations, thereby reducing unnecessary energy dissipation and muscle fatigue, and ultimately enhancing the efficiency of explosive movements [27]. This finding is highly consistent with our experimental results, showing that the assistive effect of the strap is particularly pronounced during high-intensity activities such as uphill walking, stair ascent, and squatting.

Furthermore, this assistive effect may also stem from neural modulation. Research suggests that pressure stimuli applied to the skin can enhance proprioception and neuromuscular control, thereby improving movement coordination and preventing injuries [28]. This hypothesis was partially confirmed in our experiments: one participant subjectively reported a clearer perception of muscle exertion when wearing the strap during uphill walking, while three other subjects expressed similar sentiments. This enhancement of neuromuscular feedback triggered by external tactile stimulation may be another important mechanism by which the strap improves athletic performance.

A comparison of the assistive effects between this study and other rigid and flexible exoskeleton devices is presented in Table 3.

Although the device was worn unilaterally, the risk of significant compensatory gait mechanisms was considered low. Unlike traditional knee braces that restrict joint range of motion and have been shown to increase load on the contralateral limb [29], our device was designed to avoid constraining the knee joint angle. Furthermore, the metabolic rate results provide objective evidence against the hypothesis of asymmetric compensation. Biomechanical research establishes that compensatory gait patterns significantly increase energy expenditure, potentially up to twice the normal level [29]. Our experimental data showed a significant decrease in metabolic rate when subjects wore the device. This physiological outcome contradicts the presence of forced force-shifting to the other leg, as such asymmetry would logically lead to increased, rather than decreased, metabolic cost. Therefore, the unilateral configuration does not compromise the validity of the results.

Another core finding of this study is that the influence of air bladder pressure on biomechanical benefits exhibits significant duality and dependence on motion patterns. We found that the optimal pressure for optimizing whole-body energy efficiency (reducing net metabolic rate) was inconsistent with the optimal pressure for reducing local muscle load (reducing rectus femoris RMS). This phenomenon reveals the complexity of the bladder assistance mechanism: higher pressure (30 kPa) may primarily attenuate non-functional muscle vibrations, thereby more effectively reducing the total work performed by the body on a macroscopic level, which significantly lowered metabolic rates during high-intensity activities such as jogging, uphill walking, and stair ascent. However, at the microscopic level, excessive pressure may impose over-constraint on the muscles or alter their force generation patterns, resulting in an effect on reducing muscle activity that is inferior to that of 20 kPa in certain activities.

At the same time, the assistive effect of the air bladder is highly dependent on the specific motion pattern. During low-intensity walking, the bladder had a minor impact, which may be attributed to the interference of the intervention with the body’s natural and efficient energy utilization mechanisms. Conversely, in activities requiring substantial muscular work, such as uphill walking, stair ascent, and squatting, the benefits of the bladder were maximized, particularly in reducing RF activity.

Collectively, these findings underscore the limitations of static pressure settings and point the way for the design of future intelligent assistive systems. An ideal system should not be static but adaptive. It should be capable of dynamically adjusting to the optimal pressure value based on the motion pattern recognized in real-time, as well as the user’s specific needs (such as rehabilitation load reduction or athletic performance enhancement).

Our study confirms that a lightweight deep learning model deployed on the STM32 platform enables high-accuracy real-time recognition of multiple motion patterns using only a single pressure sensor. This result first reveals the significant potential of thigh pressure signals as features for motion recognition. We speculate that the underlying reason lies in the periodic volume changes of various muscle groups under different motion patterns, which provide the model with rich discriminative information. Meanwhile, the recognition accuracy of up to 99.17% fully validates the rationality of our designed CNN-LSTM hybrid architecture: this architecture effectively synchronizes the extraction of local spatial features and temporal sequence dependencies from the pressure signals.

It is worth noting that the LSTM framework is commonly used in language models to process long-sequence dependencies, such as resolving polysemy ambiguities based on context. Similarly, our pressure data faces a challenge of “polysemy”—where the single sensor signal couples activity information from multiple muscle groups in the distal thigh. The LSTM model we adopted, with its powerful temporal modeling capability, effectively achieves the decoupling of this coupled information, thereby accurately identifying different motion patterns.

**Figure 14:**
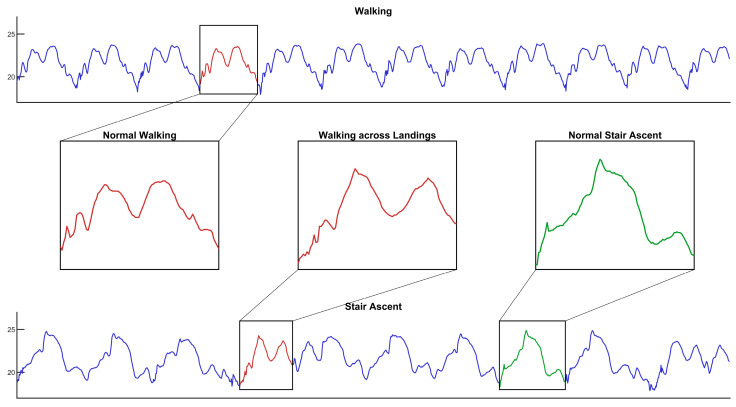
Detailed comparison of pressure waveforms between level walking and stair ascent. The magnified insets highlight the key differences and similarities. The “Walking across Landings” segment, which is part of the stair ascent sequence, exhibits a pressure pattern (red) that is highly similar to the “Normal Walking” pattern (red). In contrast, the “Normal Stair Ascent” pattern (green) shows a distinct pressure surge, differentiating it from walking.

Although the overall performance of the model is excellent, the confusion matrix indicates that primary misclassifications occur between “stair ascent” and “walking”. A review and analysis of the data acquisition process reveal that this is not a limitation of the model’s capability, but rather an issue of data label noise. Specifically, during the “stair ascent” experiments, participants had to walk across the landings between flights of stairs, where the motion pattern was effectively normal walking. These “walking” data segments, incorrectly labeled as “stair ascent”, naturally possess pressure characteristics highly similar to the “walking” category. We deduce that this portion of contaminated label data is the main reason for the model misclassifying some “stair ascent” samples as “walking”. This hypothesis is strongly supported by the visual evidence in Figure 14. As depicted, the pressure waveform for stair ascent is composed of two distinct types of cycles: one that closely resembles the pattern of level walking, and another that is characteristic of stair climbing. This finding also indirectly confirms that the model’s learning of feature patterns is accurate and sensitive. Compared to complex systems relying on multiple sensors, achieving this performance with a single sensor and only 9104 parameters offers significant advantages in terms of cost, power consumption, and deployment convenience.

Fundamentally, the high-precision recognition of locomotion modes and the significant physiological benefits of pneumatic assistance, though validated independently in this study, are intrinsically interconnected. The accurate identification of motion states serves as a prerequisite for intelligent decision-making, while pressurized actuation provides the necessary means for physical intervention. The successful validation of both functionalities within a unified hardware architecture demonstrates the feasibility of evolving this system into a closed-loop control framework. Consequently, our future work will focus on integrating these two modules to develop a mode-adaptive control strategy based on multi-objective optimization.

The core control logic is designed as a two-stage process: first, given the observed trade-off between metabolic reduction and muscle unloading, a multi-objective optimization algorithm will be employed offline to determine the optimal bladder configuration for each specific motion mode. The objective function will minimize a combined cost reflecting both metabolic and muscular outcomes, allowing the system to generate personalized profiles tailored to different user needs, such as prioritizing metabolic efficiency for athletes or muscle load reduction for rehabilitation patients. Second, during online operation, the optimized parameters will be stored in a lookup table. The system maps the recognized motion state to the pre-calculated optimal configuration in real-time.

This “offline-optimization, online-lookup” architecture ensures that the system can dynamically regulate assistance with minimal computational latency, effectively bridging the gap between motion recognition and physiological intervention.

## 5. Conclusions

This study successfully designed and validated a “sensing-actuation integrated” intelligent pressurized thigh band system, providing an effective solution to address the limitations of traditional sEMG sensing and to achieve user-friendly intelligent assistance.

At the “actuation” level, we demonstrated the significant benefits of soft pneumatic assistance. Experiments revealed a dual mechanism: a pressure of 30 kPa optimizes whole-body energy efficiency, while 20 kPa more effectively reduces local muscle load, with effects varying dynamically across motion patterns. This validates the immense potential of air bladders as lightweight, low-cost, and joint-friendly actuators for improving athletic performance.

At the “sensing” level, we successfully validated the core hypothesis that dynamic circumferential thigh pressure can serve as a reliable proxy for motion intent. The lightweight CNN-LSTM model we proposed achieved a motion recognition accuracy of 99.17% using only a single pressure signal. Furthermore, the model’s minimal resource footprint on the STM32 platform demonstrates the feasibility of achieving real-time, autonomous control strategies on resource-constrained wearable devices, laying the foundation for future adaptive systems.

In summary, this study not only provides key technical validation and a theoretical basis for the development of low-cost, adaptive intelligent assistive devices suitable for daily exercise, but also offers a new approach for the field of wearable robotics to explore alternatives to dependence on traditional complex sensors.

## Figures and Tables

**Table 1:** Classification performance overview showing accuracy, precision, recall, and F1-score for each class on the test set.

	Precision	Recall	F1-Score	Support
Uphill	1.0000	1.0000	1.0000	200
Jogging	1.0000	0.9950	0.9975	200
Squatting	0.9950	1.0000	0.9975	200
Stairs Ascent	0.9847	0.9650	0.9747	200
Standing	0.9950	0.9950	0.9950	200
Walking	0.9755	0.9950	0.9851	200
Accuracy			0.9917	1200

**Table 2:** Effects of pneumatic assistance on metabolic rate and muscle activity.

Motion Category	Metabolic Rate Reduction (30 kPa)	Muscle Activity Reduction (20 kPa)
Walking	n.s. ^1^	25.4%
Jogging	7.8%	11.1%
Uphill Walking	10.0%	29.5%
Stair Ascent	16.0%	21.6%
Squatting	N/A ^2^	29.0%

^1^. n.s. indicates no significant difference. ^2^. N/A indicates that the squat exercise is not suitable for collecting metabolic rate data.

**Table 3:** Comparison between the proposed device and representative exoskeleton studies regarding weight and effectiveness.

	System Category	Weight	Assistive Effect
M-BLUE modular exoskeleton [5]	Rigid exoskeleton	2.8 kg	Quadriceps muscle activation peak ↓^1^ 27.8%
Hip and knee lower limb assistive exoskeleton [6]	Rigid exoskeleton	12.67 kg	Rectus femoris muscle activity ↓30–38%
Bio-inspired knee soft exoskeleton system [10]	Tensile robotic suits	N/A ^2^	Knee joint torque ↓10.92%
			Knee joint power ↓30.1%
Knee exoskeleton suit [11]	Tensile robotic suits	1.72 kg	Knee joint biomechanical force ↓23.2%
Soft inflatable knee exoskeleton [15]	Expansive robotic suits	N/A	Rectus femoris muscle activation ↓7%
Soft inflatable exoskeleton [16]	Expansive robotic suits	345 g	Quadriceps surface EMG signal ↓28.52%
This study	Pressurized band	150 g	Metabolic rate ↓16%
			Rectus femoris muscle activity RMS ↓29.5%

^1^ ↓ indicates a decrease. ^2^ N/A denotes that the system weight was not reported in the literature.

## Data Availability

The data presented in this study are available on request from the corresponding author due to privacy and ethical restrictions concerning the participants.

## Abbreviations

The following abbreviations are used in this manuscript:

sEMG	Surface electromyography
IMU	Inertial measurement unit
PCB	Printed circuit board
MCU	Microcontroller unit
FPU	Floating point unit
TPU	Thermoplastic polyurethane
RF	Rectus femoris
MVC	Maximal voluntary contraction
RMS	Root mean square
SD	Standard deviation
MACC	Multiply-accumulate operations
